# Mr. Thomas Windsor

**Published:** 1910-04-30

**Authors:** 


On April 13, Mr. Thomas Windsor died at his home, Great Budworth, in Cheshire, at the age of seventy-eight. In 1853 Mr. Windsor became an M.R.C.S., and in the following year an L.S.A.

